# Regulation of colony stimulating factor-1 expression and ovarian cancer cell behavior *in vitro* by miR-128 and miR-152

**DOI:** 10.1186/1476-4598-11-58

**Published:** 2012-08-21

**Authors:** Ho-Hyung Woo, Csaba F László, Stephen Greco, Setsuko K Chambers

**Affiliations:** 1 Arizona Cancer Center, University of Arizona, Tucson, AZ 85724, USA

**Keywords:** miR-128, miR-152, CSF-1 mRNA, Post-transcriptional regulation, motility and adhesion

## Abstract

**Background:**

Colony stimulating factor-1 (CSF-1) plays an important role in ovarian cancer biology and as a prognostic factor in ovarian cancer. Elevated levels of CSF-1 promote progression of ovarian cancer, by binding to CSF-1R (the tyrosine kinase receptor encoded by *c-fms* proto-oncogene).

Post-transcriptional regulation of CSF-1 mRNA by its 3’ untranslated region (3’UTR) has been studied previously. Several *cis*-acting elements in 3’UTR are involved in post-transcriptional regulation of CSF-1 mRNA. These include conserved protein-binding motifs as well as miRNA targets. miRNAs are 21-23nt single strand RNA which bind the complementary sequences in mRNAs, suppressing translation and enhancing mRNA degradation.

**Results:**

In this report, we investigate the effect of miRNAs on post-transcriptional regulation of CSF-1 mRNA in human ovarian cancer. Bioinformatics analysis predicts at least 14 miRNAs targeting CSF-1 mRNA 3’UTR. By mutations in putative miRNA targets in CSF-1 mRNA 3’UTR, we identified a common target for both miR-128 and miR-152. We have also found that both miR-128 and miR-152 down-regulate CSF-1 mRNA and protein expression in ovarian cancer cells leading to decreased cell motility and adhesion *in vitro*, two major aspects of the metastatic potential of cancer cells.

**Conclusion:**

The major CSF-1 mRNA 3’UTR contains a common miRNA target which is involved in post-transcriptional regulation of CSF-1. Our results provide the evidence for a mechanism by which miR-128 and miR-152 down-regulate CSF-1, an important regulator of ovarian cancer.

## Background

The colony stimulating factor-1 (CSF-1) and its receptor CSF-1R (encoded by the *c-fms* proto-oncogene) comprise a reciprocal system that has been previously linked to several human epithelial cancers including ovarian, breast, and prostate cancers [1-4]. Besides its critical role in macrophage differentiation and proliferation [5], CSF-1 and *c-fms* are also key players in bone metabolism [6] and female reproduction [7]. In ovarian cancer, CSF-1 also has an important role as a biomarker and prognostic factor, as high levels of this protein were linked to poor patient outcome [8,9]. CSF-1 was also associated with cancer virulence by having the capacity to augment the invasive ability of human ovarian cancer cells [10], and by promoting metastasis [11].

CSF-1 has several alternatively spliced transcripts that encode for different sizes of CSF-1 proteins with different functionality [12]. Its biological function as a cytokine in autocrine and paracrine signaling is achieved mostly by a secreted form that is the product of a 3,939nt transcript excluding poly A^+^ tail [13]. This transcript contains a long, 2,172nt 3’UTR. In ovarian cancer cells, a major unprocessed CSF-1 of 60.1 kDa polypeptide is produced by the 3,939nt transcript. This monomer is processed further by glycosylation and forms an over 200 kDa homodimeric glycoprotein which is the most abundant form of secreted CSF-1 in ovarian cancer [14,15].

Among the CSF-1 regulatory events, major importance is attributed to CSF-1 post-transcriptional regulation achieved by mRNA 3’UTR binding factors. Previously, we identified GAPDH protein which binds to ARE and stabilizes CSF-1 mRNA leading to post-transcriptional up-regulation of CSF-1 in ovarian cancer cells [16].

MicroRNAs (miRNAs) are small single-strand RNAs of 21–23 nucleotides in length that regulate several biological functions (i.e., differentiation, hematopoiesis, tumorigenesis, apoptosis, development, and cell proliferation) through modulating the stability and/or translation efficiency of target mRNAs [17]. They are predicted to regulate about 60% of mammalian mRNAs [18]. It has been found that mRNAs with long 3’UTRs are more susceptible to miRNA regulation than those with short 3’UTRs as the latter ones lack in number of binding sites necessary for multiple miRNA binding and regulation [19].

Although previous studies have reported miRNA regulation of CSF-1, most of these describe indirect regulation through additional miRNA targeted proteins in non-ovarian cells [20]. To the best of our knowledge, there are only two previous reports of a miRNA that shows direct CSF-1 regulatory abilities in an ovarian system [21,22].

We predict that, since the 3,939nt CSF-1 transcript has a vast (2,172nt) 3’UTR, miRNAs may play an important regulatory role in mediating the cellular levels and biological functions of CSF-1 in ovarian cancer. In this report, we study 3’UTR targets for binding miRNAs, and find that both miR-128 and miR-152 down-regulate CSF-1 expression in ovarian cancer. Our goal is to identify miRNAs that down-regulate CSF-1 expression, and eventually open an avenue for possible treatment options for ovarian carcinomas.

## Results

### Bioinformatics analysis of potential miRNAs targeting CSF-1 mRNA 3’UTR

To assess the most common miRNA target sequences located in the 3’UTR of the 3,939nt CSF-1 mRNA, we used the MirWalk text-mining algorithm [23] applied to the mirBase-15 database [24]. This search engine uses its own algorithm to find putative miRNA binding sites for a gene of interest and also compares its findings with a number of other search tools (i.e., miRanda, miRDB, miRWalk, PicTar, PITA, RNA22 and TargetScan/TargetScanS (version 5.1) [18,23,25-31]. This search reveals the putative target sequence ‘^2573^CACUG^2577^’ which has the most hits with 14 miRNAs having at least a hit number of 4 (miR-27a/b, -128, -130a/b, -135a/b, -148a/b, -152, -214, -301a/b, -454) (Table 1). Among these miRNAs, we focused on 7 miRNAs, or 50%, of these miRNAs. Selected miRNAs for further analysis in this report are miR-152, -128, -27a, -214, -454; with results concerning the role of miR-130a and miR-301a in another context to be reported elsewhere (Woo *et al*. unpublished).

**Table 1:** Summary of bioinformatics analysis of 14 miRNAs targeting CSF-1 mRNA 3’UTR, having hit numbers ≥4

	**miRANDA**	**miRDB**	**miRWalk**	**PICTAR4**	**PITA**	**RNA22**	**Targetscan**	**Sum**
**miR-152**	1	0	1	1	1	1	1	**6**
**miR-128**	1	1	1	0	1	0	1	**5**
**miR-27a**	1	0	1	1	1	1	0	**5**
**miR-27b**	1	0	1	1	1	1	0	**5**
**miR-214**	1	1	0	1	1	0	1	**5**
**miR-130a**	1	0	1	1	1	1	0	**5**
**miR-130b**	1	0	1	1	1	1	0	**5**
**miR-301a**	1	1	1	0	1	0	0	**4**
**miR-301b**	1	1	1	0	1	0	0	**4**
**miR-454**	1	0	1	1	1	0	0	**4**
**miR-135a**	1	0	1	1	1	0	0	**4**
**miR-135b**	1	0	1	1	1	0	0	**4**
**miR-148a**	1	0	1	1	1	0	0	**4**
**miR-148b**	1	0	1	1	1	0	0	**4**

1 = hit, 0 = no hit.

### Expression of miR-128, miR-152, miR-27a, miR-214, and miR-454 in ovarian cancer cells

**Figure 1:**
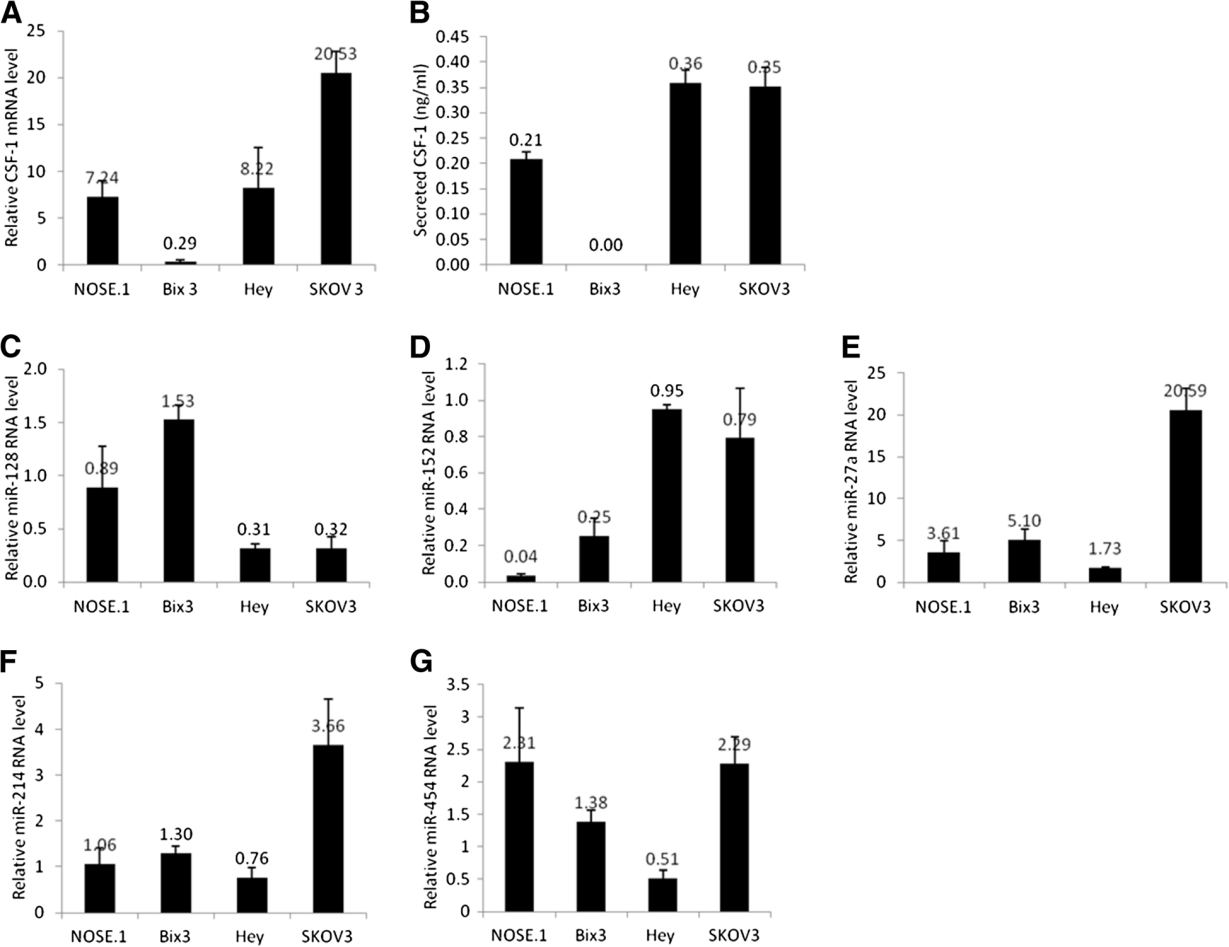
**A)Expression of CSF-1 mRNA in ovarian cancer cells. B**) Secreted CSF-1 protein levels detected by ELISA. **C**) miR-128 RNA level is high in Bix3 ovarian cancer cells compared to Hey and SKOV3 ovarian cancer cells. **D**) miR-152 RNA level is low in less aggressive Bix3 cells compared to Hey and SKOV3 cells. **E**) miR-27a RNA level in ovarian cancer cells. **F**) miR-214 RNA level in ovarian cancer cells. **G**) miR-454 RNA level in ovarian cancer cells. Bars represent mean ± SD of triplicate experiments.

To correlate selected miRNA expression patterns with CSF-1 mRNA and protein expression, we used three ovarian cancer cell lines and NOSE.1 ovarian epithelial cells which are minimally invasive [32]. Bix3 ovarian cancer cells are less invasive compared to the metastatic and more invasive ovarian cancer cells, Hey and SKOV3 [10]. Bix3 also express a low level of CSF-1 compared to Hey and SKOV3, which express high levels of CSF-1 mRNA and also secreted CSF-1 protein (Figure 1A, B). In addition, NOSE.1 express intermediate amounts of CSF-1 mRNA and secreted CSF-1 protein, and was used for comparison of miRNA expression. Expression of miR-128 is significantly inversely correlated with CSF-1 protein expression (correlation coefficient = −0.998, p = 0.002), with miR-128 RNA level low in Hey and SKOV3 and high in Bix3 ovarian cancer cells, as well as in NOSE.1 ovarian cells (Figure 1C). In contrast, expression of miR-152 appears to be positively correlated with CSF-1 expression, with miR-152 expressed at a high level in Hey and SKOV3 with lower expression seen in Bix3 cells and minimal expression in NOSE.1 cells (Figure 1D), but this correlation was not statistically significant. The expression patterns of the remaining 3 miRNAs were more variable. miR-27a and miR-214 are expressed highly in SKOV3 cells compared to other cell lines (Figure 1E, F). miR-454 is expressed more in SKOV3 and NOSE.1 cells than Hey and Bix3 cells (Figure 1G). Their expression patterns were not significantly correlated with CSF-1 mRNA or protein expression (P = NS).

Since generally miRNAs are known to down-regulate target mRNAs, we would expect that CSF-1 mRNA and miRNA levels would be inversely correlated. However, there are also reports that some miRNAs are involved in up-regulation of translation [33]. This usually occurs through a feedback loop when the target mRNA is indirectly regulated by upstream inducers or inhibitors. To address this issue, we decided to study, in more detail, the roles of miR-128 (as being inversely correlated to CSF-1 expression) and miR-152 (as appearing to be most positively correlated to CSF-1 expression) in the post-transcriptional regulation of CSF-1 expression.

### Expression pattern between host genes and miRNAs correlate with each other

**Figure 2:**
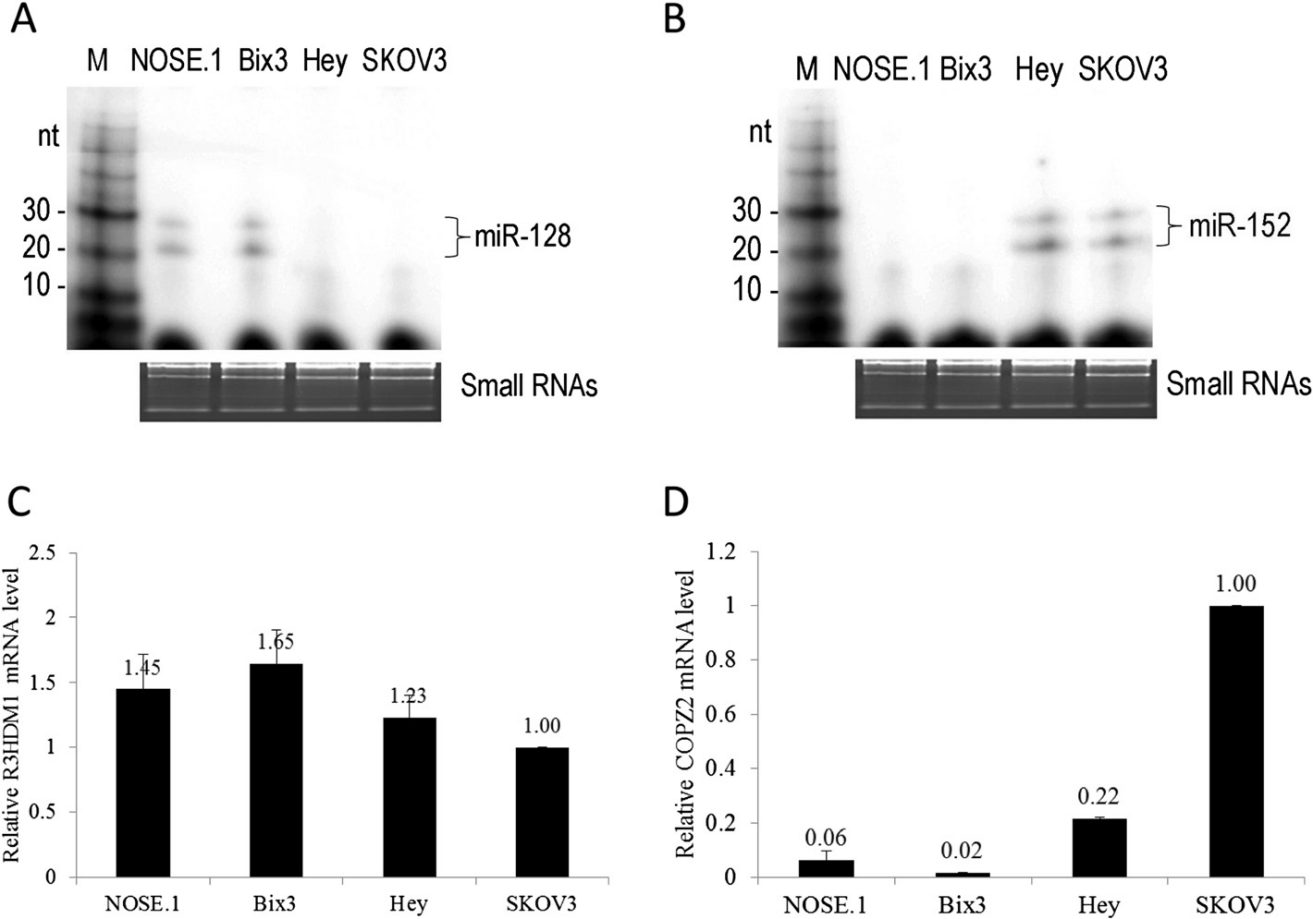
**A)Splinted ligation of miR-128 in ovarian cancer cells.** miR-128 RNA is detected in both NOSE.1 and Bix3 cells. **B**) Splinted ligation of miR-152 in ovarian cancer cells. miR-152 RNA is detected in both Hey and SKOV3 cells. **C**) miRNA expression pattern follows “host” gene expression pattern. R3HDM1 mRNA expression in ovarian cancer cells. **D**) COPZ2 mRNA expression in ovarian cancer cells. GAPDH mRNA was used as a loading control in qRT-PCR. Bars represent mean ± SD from triplicate experiments.

To further confirm the expression pattern of miR-128 and miR-152 in ovarian cancer cells, we applied the Splinted Ligation technique to directly detect these miRNAs [34]. miR-128 RNA is detected in both NOSE.1 and Bix3 cells and not detectable in Hey and SKOV3 ovarian cancer cells (Figure 2A). In contrast, miR-152 RNA is detected in Hey and SKOV3 cells, and not detectable in NOSE.1 and Bix3 cells (Figure 2B). These results correlate well with qRT-PCR data (Figure 1C, D).

Since profiling of host mRNA expression could be correlated to the expression pattern of microRNAs, the knowledge of the relative expression pattern of miRNAs and their “host” genes would allow for a better profiling of the miRNA expression. This aspect could be important when using the expression of a certain miRNA as a biomarker to correlate with disease outcome. miR-128 is highly expressed in human brain tissue and its function is linked to neuronal differentiation [35]. miR-128 gene is imbedded in two paralogous genes, both present in the intronic region of their respective “host” genes. miR-128-1 is in R3HDM1 gene on chromosome 2q21.3 and miR-128-2 is in ARPP21 gene on chromosome 3p22*.* Both gene products are processed into the same mature miR-128 [36]. miR-152 belongs to the miR-148 family whose putative role is still elusive, but it has been studied in hepatic [37], cervical [38], and brain cancers [39]. miR-152 gene is imbedded in the intronic region of COPZ2 gene, which is a subunit of coatomer protein complex 1 (COP1) known to be responsible for Golgi to ER transport [40].

In both cases, expressions of miR-128 and miR-152 follow their host gene expression patterns (Figure 2C, D). The slight discrepancies between host gene and miRNA expression pattern may be due to differential processing that is involved during the maturation of mRNAs and miRNAs.

### CSF-1 mRNA 3’UTR is a direct target for miR-128 and miR-152 in ovarian cancer cells

**Figure 3:**
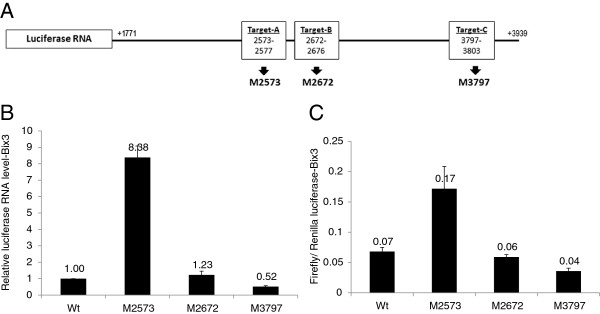
**A) Luciferase reporter constructs with 2,172nt full length CSF-1 mRNA 3’UTR cloned at the 3’-end of the luciferase mRNA.** Either CSF-1 mRNA 3’UTR wild type, Target-A point-mutation (M2573), Target-B point-mutation (M2672), or Target-C point-mutation (M3797) sequences were cloned. **B**) Only Target-A construct results in an increase in luciferase RNA and **C**) luciferase activity in Bix3 cells. Bars represent mean ± SD from triplicate experiments.

The miRanda v3.0 target scanning algorithm predicts two target regions (Target-A and –C) for miR-128 and two target regions (Target-A, and -B) for miR-152 in the 2,172nt CSF-1 mRNA 3’UTR (Figure 3A). All three targets share the common sequence of ‘CACUG’. These two miRNAs are related through a Target-A sequence at region 2573–2577. This Target-A sequence appears to be important for CSF-1 regulation by miRNAs because it is predicted to be a target sequence for several miRNAs. Moreover, the ‘CACUG’ comprising these target sequences was described as a conserved target motif that conferred miRNA regulation to its mRNA 3’UTR region in mouse embryonic stem cells [41].

To find the actual target sequence for miR-128 and miR-152, we used a luciferase reporter system. A full length wild type 2,172nt CSF-1 mRNA 3’UTR was ligated at the 3’-end of luciferase RNA. In addition, the three individual target sequences within the wild type 3’UTR sequence were individually point-mutated from ‘CACUG’ to ‘CGCGC’ and ligated to the 3’-end of luciferase RNA (Figure 3A). These constructs were transfected into Bix3 cells. Only the Target-A mutation (M2573 construct) increased both luciferase RNA level by 8.38-fold (p < 0.001) and luciferase activity by 2.43-fold (p < 0.001) compared to wild type sequence (Figure 3B, C). There was no significant difference (p = NS) between the effects of Target-B mutation, Target-C mutation, or the wild type sequence. This data suggests that Target-A is a *cis*-acting regulatory sequence in CSF-1 mRNA 3’UTR which may respond to miRNAs. This data also suggests that both miRNAs have more effects at the RNA than protein levels.

**Figure 4:**
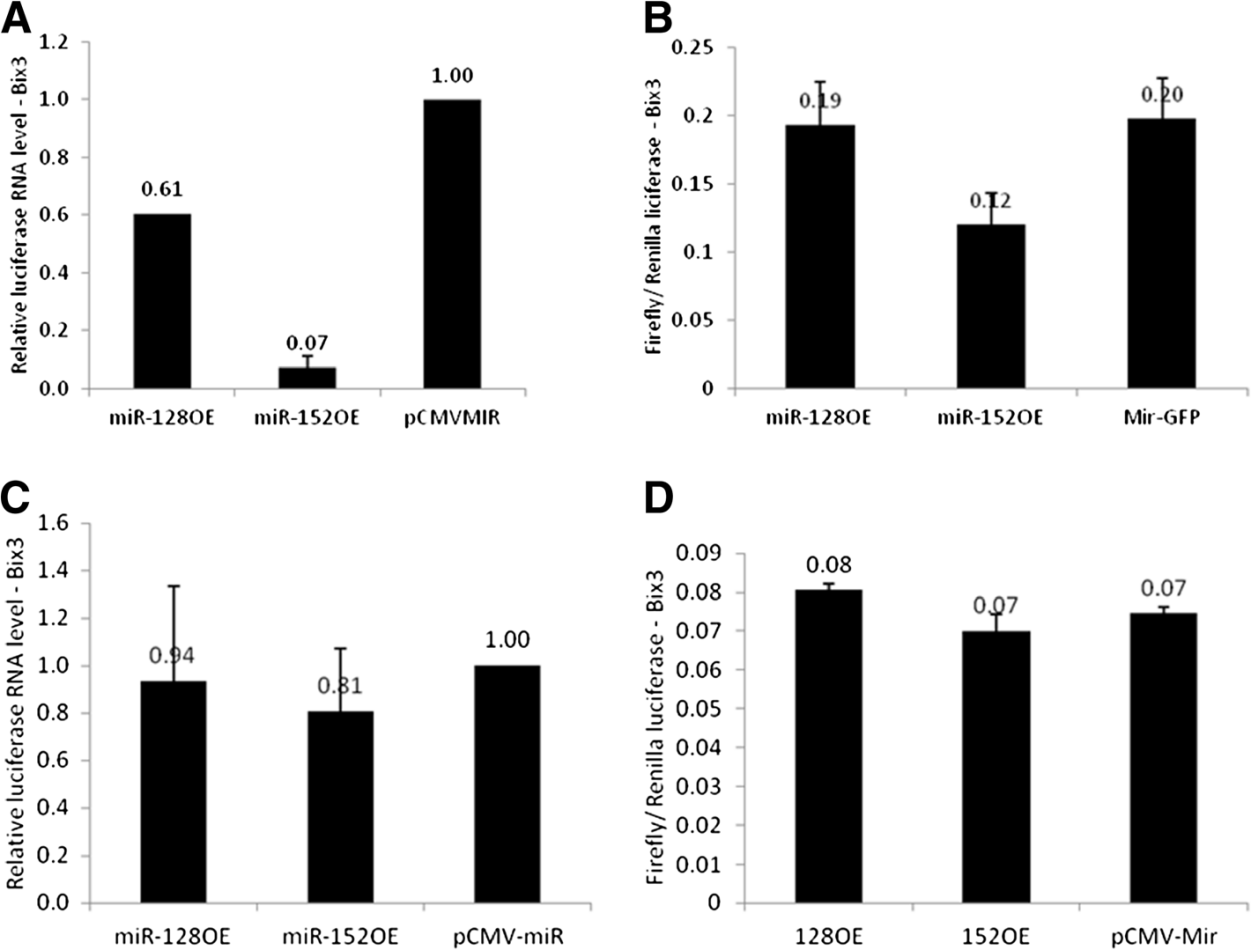
**Target-A is an active target for miR-128 and miR-152.** Either miR-128 or miR-152 was overexpressed together with either **A, B**) wild type construct (Luc-CSF-1 3’UTR-Wt) or **C, D**) Target-A mutant construct (Luc-CSF-1 3’UTR-Mut) in Bix3 cells. Bars represent mean ± SD from triplicate experiments.

To further confirm Target-A as an actual miRNA-responding sequence, miR-128 or miR-152 was overexpressed together with either wild type construct (Luc-CSF-1 3’UTR-Wt) or Target-A mutant construct (Luc-CSF-1 3’UTR-Mut). Overexpression of either miR-128 or miR-152 in Bix3 cells co-transfected with the wild type construct decreased luciferase RNA by 39% and 93%, respectively (p < 0.001). Luciferase activity was decreased by 40% in response to miR-152 overexpression (p = 0.006) (Figure 4A,B). Overexpression of miR-128 in these cells already overexpressing high levels of endogenous miR-128 (Figure 1C), does not change luciferase activity (p = NS). In this context, exogenously introduced miR-128 may not have a strong influence on luciferase translation. In contrast, overexpression of either miR-128 or miR-152 in Bix3 cells transfected with the Target-A mutant construct did not decrease luciferase RNA significantly (p = NS) compared to the wild type construct (Figure 4C). In the presence of the Target-A mutant construct, miR-128 overexpression also had no significant effect (p = NS) on luciferase activity. There was a small but statistically significant (p = 0.03) decrease in luciferase activity by miR-152 overexpression (Figure 4D). Comparison of Figures 4B and 4D, however, demonstrate that the Target-A mutation construct largely attenuates the effect of miR-152 overexpression on luciferase activity. This suggests that both miRNAs target this region Target-A (^2573^CACUG^2577^) in CSF-1 mRNA 3’UTR.

### miR-128 and miR-152 down-regulate CSF-1 mRNA and protein expression

**Figure 5:**
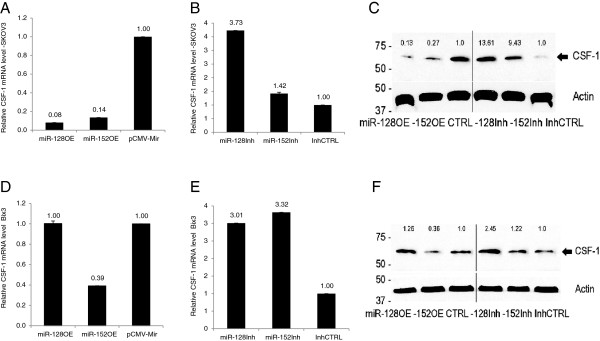
**miR-128 and miR-152 inhibit CSF-1 expression in SKOV3 and Bix3 cells.** Either SKOV3 or Bix3 cells were transfected with the **A, D)** miR-128 or miR-152 overexpression plasmids (miR-128OE, miR-152OE); or **B, E)** inhibitor plasmids (miR-128Inh, miR-152Inh); or vector controls. **C, F**) CSF-1 protein (~60 kDa) levels in SKOV3 and Bix3 cells were detected by immunoblot analysis. mRNA and protein expression levels were normalized by the levels of GAPDH mRNA and actin, respectively. Bars represent mean ± SD from triplicate experiments.

To determine the effects of miR-128 and miR-152 on the expression of CSF-1, either miR-128 or miR-152 were over-expressed or inhibited in SKOV3 and Bix3 ovarian cancer cells. In SKOV3, overexpression of miR-128 decreased CSF-1 mRNA level by 92% (p < 0.001) (Figure 5A) and CSF-1 protein levels (~60 kDa) by 87% (Figure 5C). In SKOV3, overexpression of miR-152 decreased CSF-1 mRNA level by 86% (p < 0.001) (Figure 5A) and CSF-1 protein levels (~60 kDa) by 73% (Figure 5C). In contrast, in SKOV3, inhibition of miR-128 increased CSF-1 mRNA level by 3.73-fold (p < 0.001) (Figure 5B) and CSF-1 protein level by 13.61-fold (Figure 5C). In SKOV3, inhibition of miR-152 increased CSF-1 mRNA level by 1.42-fold (p < 0.001) (Figure 5B) and protein level by 9.43-fold (Figure 5C). Alteration of both miRNA levels showed more effect on CSF-1 protein levels than mRNA levels in SKOV3.

In Bix3 cells, the miRNA effect is less significant than in SKOV3 cells. Overexpression of miR-128 does not alter CSF-1 mRNA (p = NS) or protein levels (Figure 5D, F). Since endogenous miR-128 RNA level is already high in Bix3 (Figure 1), overexpression by exogenously introduced miR-128 RNA may have little effect on CSF-1 expression. In contrast, inhibition of miR-128 increased CSF-1 mRNA level by 3.01-fold (p < 0.001) (Figure 5E) and CSF-1 protein level (~60 kDa) by 2.45-fold (Figure 5F). In Bix3, overexpression of miR-152 decreased CSF-1 mRNA level by 61% (p < 0.001) (Figure 5D) and protein levels by 64% (Figure 5F). In contrast, inhibition of miR-152 increased CSF-1 mRNA level by 3.32-fold (p < 0.001) (Figure 5E) and CSF-1 protein level (~60 kDa) by 1.22-fold (Figure 5F). Either overexpression or inhibition of miR-152 has effect on both CSF-1 mRNA and protein levels.

While some differential effects of miRNA alteration are seen between the different ovarian cancer cell lines, overall, the effect of miR-128 and −152 on down-regulation of CSF-1 expression are similar, and may be achieved by both translational repression and mRNA decay. This down-regulation of CSF-1 mRNA by both miRNAs is observed in the context of differing miRNA expression patterns in the ovarian cancer cells.

### miR-128 and miR-152 inhibit cellular motility and adhesion of ovarian cancer cells

It has been previously established that CSF-1 imparts invasiveness and metastasis in epithelial ovarian cancer [8,10,11]. We have shown that this is due, at least in part, to CSF-1 regulation of uPA, a well-known marker of invasiveness in ovarian cancer [10]. It was also recently revealed that both miR-128 and miR-152 have the ability to inhibit neuroblastoma cell motility and invasiveness when overexpressed [39].

**Figure 6:**
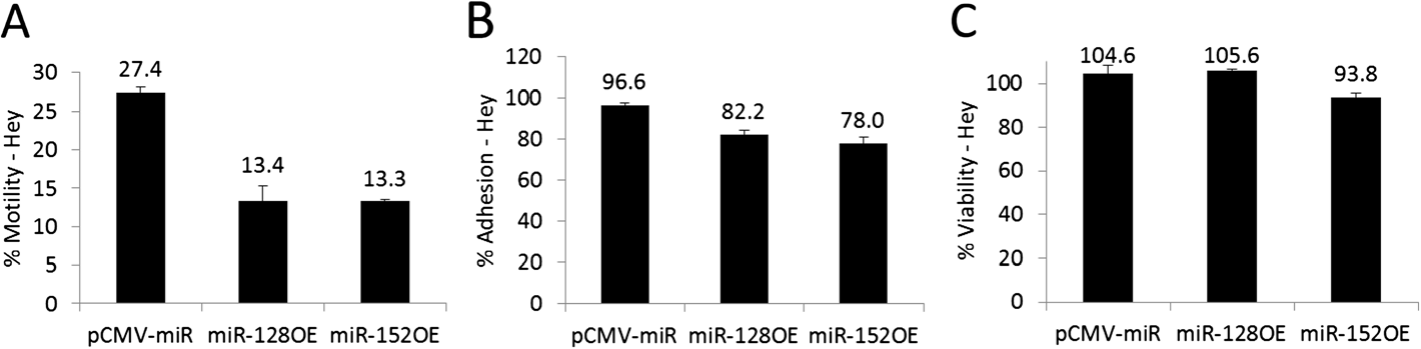
**Ovarian cancer cell adhesiveness and motility is inhibited by miR-128 and miR-152.** After transfection with, miR-128 overexpression construct (miR-128OE), miR-152 overexpression construct (miR-152OE), or Empty vector (pCMV-miR), Hey cells were plated on **A**) an 8 micron pore membrane for a 6 hour directed motility assay. **B**) Human matrix-coated membrane for a 2 hour adhesion assay. **C**) Viability of miRNA overexpression construct transfected Hey cells 24 hours post transfection was determined by a cell proliferation assay.

We studied the ability of Hey ovarian cancer cells to either adhere to a human derived simple matrix-coated plate in an adhesion assay or translocate through an 8 μm pore-size membrane towards chemo-attractants in a motility assay. The motility of Hey cells was significantly curtailed by over 50% by the overexpression of either miR-128 or miR-152 (p < 0.001) (Figure 6A). Furthermore, overexpression of miR-128 or miR-152 in Hey cells inhibited cell adhesiveness by 15-20% (p < 0.001) (Figure 6B). At the same time, overexpression of miR-128 had no significant effect on viability (p = NS) (Figure 6C). There was a small effect by overexpression of miR-152 on viability (p = 0.004) (Figure 6C).

Our findings demonstrate that both miR-128 and miR-152 can negatively impact cell motility and adhesiveness of human ovarian cancer cells, important aspects of their metastatic potential, correlated with suppression of CSF-1 expression.

## Discussion

CSF-1 is an established regulator of ovarian cancer biology [8-11], imparting invasiveness and metastasis [9,11], making it a potentially appropriate therapeutic target. The relatively long 3’UTR of CSF-1 mRNA makes the 3’UTR a likely target for post-transcriptional regulation. We have been studying the effect of GAPDH protein on CSF-1 mRNA stability. GAPDH binds to the AREs in CSF-1 mRNA 3’UTR and stabilizes CSF-1 mRNA. Down-regulation of GAPDH by siRNA decreases CSF-1 expression in ovarian cancer cells [16].

In the present study, our goal was to identify miRNAs that down-regulate CSF-1 expression, a small step in our overall quest to find specific inhibitors which may ultimately impact on ovarian cancer metastasis. By using *in silico* text-mining algorithms against the CSF-1 mRNA 3’UTR, we selected miR-128 and miR-152 that would fit the profile of having regulatory abilities of CSF-1. While miR-128 and miR-152 possess target sequences in the CSF-1 mRNA 3’UTR, their expression patterns in the ovarian cancer cell lines proved to be different. miR-128 RNA level is lower in the invasive, metastatic Hey and SKOV3 ovarian cancer cells in comparison to the less invasive and tumorigenic Bix3 ovarian cancer cells (Figure 1C). In contrast, miR-152 level was lower in the Bix3 cells than in the Hey and SKOV3 ovarian cancer cells (Figure 1D). Despite this difference in baseline expression pattern, we find that both miRNAs down-regulated CSF-1 mRNA and protein in ovarian cancer cells (Figure 5). A relatively small number of ovarian cancer cell lines may not give sufficient information when comparing miRNA expression patterns to effect on target mRNA (CSF-1 mRNA).

The majority of the miRNAs originate from intergenic regions far from other known genes and they possess independent transcription units. On the other hand, about a quarter of human miRNA loci are intragenic and they reside in the intronic regions of pre-mRNAs [42]. Most of these latter ones will have a preferential sense orientation with the “host gene” and because they are lacking their own promoters, as a result, will be processed from introns [43]. Sharing a common promoter can result in miRNAs and “host” genes exhibiting regulatory relationships. In our study, both miR-128 and miR-152 reside in introns of R3HDM1 gene and COPZ2 gene, respectively. Their expression patterns follow those of their host transcripts. COPZ2 encodes coatomer protein complex ζ2, which is involved in intracellular traffic and autophagy in golgi [40]. miR-152 and its host gene COPZ2 are silenced in tumor cells and introduction of miR-152 precursor inhibited tumor cell (MDA-MB-231, HeLa) growth [40]. Recently, both miR-128 and miR-152 have been shown to inhibit neuroblastoma invasiveness [39]. These data suggest important biologic roles of miR-128 and miR-152 in cancer. In this report, we add the findings that over-expression of miR-128 or miR-152 in ovarian cancer cells results in a significant reduction in both motility and adhesiveness (Figure 6), therefore inhibiting important aspects of invasiveness and metastasis.

There is a recent report stating that the predominant effect of mammalian miRNAs is on mRNA decay which results reduced translation [44]. In contrast, in zebra fish, miR-430 reduced translation initiation prior to inducing mRNA decay [45]. Djuranovic *et al.*[46] reported miRNA-mediated translational repression is followed by mRNA deadenylation. In addition, the concept of mRNA destabilization by miRNAs gained support by genome-wide observation studies [47]. In SKOV3, effects of either miR-128 or miR-152 are more prominent on CSF-1 protein level than on the CSF-1 mRNA level (Figure 5A-C). In contrast, in Bix3, both miRNAs have either a similar or slightly more influence on the CSF-1 mRNA level than CSF-1 protein level (Figure 5D-F). Different cell lines, as expected, show some differential responses to miRNAs, in part due to additional 3’UTR factors which may regulate miRNA activity. Identifications of these other regulatory factors are in progress in our laboratory.

In CSF-1 mRNA 3’UTR, we identified three potential miRNA target sequences for miR-128 and/or miR-152 (Figure 3). Target-A appears to be a miRNA ‘hot-spot’ as our bioinformatics analysis predicted at least fourteen miRNAs, including miR-128 and miR-152, targeting a region of 2573–2577 (Target-A) in CSF-1 mRNA 3’UTR (Figure 3). This Target-A sequence is highly conserved both in human and mouse [41]. Mutation of Target-A resulted in a dramatic increase in reporter RNA and activity when compared to the wild-type construct (Figure 3). Target-A mutation also abrogated response of reporter RNA and activity to miR-128 and miR-152 over-expression (Figure 4). This suggests that Target-A is a critical *cis*-acting regulatory sequence, and we have validated that it serves as a direct target for at least miR-128 and miR-152 (Figure 4).

## Conclusion

The current study identifies miR-128 and miR-152 as important regulators for CSF-1 mRNA and protein expression, and of ovarian cancer cell behavior. We identify an important CSF-1 3’UTR miRNA common target sequence through which these miRNAs function.

## Methods

### Cell culture

Human ovarian cancer cells, Bix3, SKOV3 and Hey [10,11], were grown in 10% fetal bovine serum-enriched Dulbecco's Modified Eagle/F12 Ham’s medium (Invitrogen) supplemented with 1% penicillin-streptomycin (HyClone). Immortalized ovarian epithelial cells NOSE.1 [32] were grown in M199/MCDB1051 supplemented with 15% FBS and 1% penicillin-streptomycin (HyClone). All cells were incubated at 37°C and 5% CO_2_.

### Quantitative real-time RT-PCR for miRNAs

Total RNA was extracted with Trizol (Invitrogen). miRNA expression was determined by the stem-loop qRT-PCR to increase the specificity of miRNA amplification [48]. miRNA and tRNA specific cDNA synthesis was followed by real-time PCR on an Eppendorf Realplex2 with tRNA as internal loading control. Reactions were incubated during initial denaturation for 10 min at 95°C, then for 40 cycles of 15 sec at 95°C and 1 min 60°C. Final miRNA expression values were calculated with the ΔΔC_T_ method [49]. Primer sequences used are shown in Table 2.

**Table 2:** List of primers for qRT-PCR

**miRNA-R**	**GTGCAGGGTCCGAGGT**
miR-128-F	TCCGATCACAGTGAACCGGT
miR-128-RT	GTCGTATCCAGTGCAGGGTCCGAGGTATT CGCACTGGATACGAC AAAGAG
miR-152-F	TCCGA TCAGTGCATGACAGA
miR-152-RT	GTCGTATCCAGTGCAGGGTCCGAGGTATT CGCACTGGATACGAC CCAAGT
miR-27a-F	TCCGA TTCACAGTGGCTAA
miR-27a-RT	GTCGTATCCAGTGCAGGGTCCGAGGTATT CGCACTGGATACGAC GCGGAAC
miR-214-F	TCCGA ACAGCAGGCACAGAC
miR-214-RT	GTCGTATCCAGTGCAGGGTCCGAGGTATT CGCACTGGATACGAC ACTGCCT
miR-454-F	TCCGA TAGTGCAATATTGCTTA
miR-454-RT	GTCGTATCCAGTGCAGGGTCCGAGGTATT CGCACTGGATACGAC ACCCTA
CSF-1-F	CATCTCAGCCCCACCTGCATGGTA
CSF-1-R	TCCTGGGCAGGAAGGGAAAGTC
GAPDH-F	GCAGGCGTCGGAGGGCCCCCTC
GAPDH-R	GGGACTGAGTGTGGCAGGGACTCC
G-418-F	TCAGGATGATCTGGACGAAGAGC
G-418-R	CAGCAATATCACGGGTAGCCAAC
LucE-F	AACAATCCGGAAGCGACCAACG
LucE-R	AACACAACTCCTCCGCGCAAC

Similarly, CSF-1 and Luciferase transcripts were quantified with real-time PCR using the following primer set shown in Table 2. Calculations were based on the GAPDH mRNA or G-418 RNA internal controls.

### Splinted ligation

miRNA expression was confirmed by splinted ligation as described by Maroney *et al.*[21]. In short, bridge and ligation oligonucleotides were designed for the miRNAs of interest. The ligation oligo was labeled with [γ-^32^P]-ATP (Perkin Elmer, cat. no. BLU002A) using T4 polynucleotide kinase (Fermentas). Separation of ligation mixture was performed on a 10% urea gel and radioisotope emission was detected by phosphor imager.

### Transient transfection

Cells were plated on a 6 well plate one day prior to transfection with 4 μg of plasmid DNA/well using 2.5:1 v/w ratio of Fugene HD (Promega). miRNA expression vectors and miRNA target reporter vectors were purchased from Origene.

### Immunoblotting

Fifty μg of total protein lysates were subjected to SDS-PAGE and electroblotted onto PVDF membrane. The membranes were probed with mouse monoclonal anti-CSF-1 (ab66236, Abcam) and anti-Pan Actin (ACTN05, NeoMarkers, Fremont, CA) antibodies. After TBS-T washes and incubation with anti-mouse (HRP)-conjugated secondary antibody, the proteins were detected with a SuperSignal chemiluminescence (Pierce).

### Mutation of CSF-1 mRNA 3’UTR targets

Each target was replaced by Asc I endonuclease site which converts ‘TGCACTGA’ to ‘GGCGCGCC’ by PCR cloning and fused to the 3’end of luciferase RNA in pMir-Target (Origene).

### Luciferase assay

Forty eight hours after transfection with the reporter plasmid, cells were lysed and luciferase activity was determined using the Dual Luciferase Assay System according to the manufacturerâ€™s protocol (Promega). Transfection efficiency was determined (where needed) by cotransfection with a GFP plasmid and microscopy.

### Transwell motility assay and adhesion assay

For the directed motility assay, 24 hours post transfection, 4 x 10^4^ cells were seeded in 1% Nu serum in the top chamber of 24-well inserts with uncoated 8 μm pore membranes (BD biosciences). The bottom chamber contained 20% FBS and 12.5 μg/ml fibronectin as chemo-attractants. Six hours after seeding, the top chamber cells were wiped off with Q-tips and the top and bottom chambers were washed with cold PBS, then dried and frozen at -80°C for 30 minutes. Bottom chamber cells were quantified by the lysis method using CyQuant Cell Proliferation Assay Kit (Invitrogen) as per the manufacturer’s protocol.

For the adhesion assay, 24-well inserts with human matrix-coated membranes (BD biosciences) were used. The human matrix consisted of type IV collagen, laminin, and gelatin. 24 hours post transfection, 5 x 10^4^ Hey cells were implanted and incubated for 2 hours prior to crystal violet staining according to the previous report [11].

The WST-1 assay (Clontech) was used to assess degree of cell proliferation among the conditions 24 after transfection.

### Statistical analysis

Data is depicted as mean ± SD from at least three independent experiments. The one-way ANOVA test was performed using SigmaStat (Jandel Scientific Corp.). P < 0.05 was considered statistically significant. The Pearson product moment correlation test was performed using SigmaStat (Jandel Scientific Corp.) for correlation analysis between miRNA and CSF-1 mRNA or protein expression levels.

## Abbreviations

CSF-1: Colony-stimulating factor-1; miR: microRNA; cDNA: Complementary-DNA; PCR: Polymerase chain reaction; RT-PCR: Reverse transcriptase PCR; qRT-PCR: Quantitative real time RT-PCR.

## Competing interests

The authors declare that they have no competing interests.

## Authors’ contributions

HHW and CFL carried out most of the experiments including the quantitative real-time qRT-PCR for CSF-1 mRNA, miR-128 and miR-152 as well as the overexpression and suppression of miRNAs and wrote the manuscript. SG performed the WST-1 cell proliferation assay. HHW and SKC discussed the design of the experiments, the results, the analysis, and wrote the manuscript. All authors read and approved the final manuscript.

## Grant support

This work was supported in part by NIH/NCI P30 CA23074 and NIH CA60665, Women’s Cancers of the University of Arizona Cancer Center, Tucson AZ, and the Rodel Foundation (to SKC.).

## References

[B1] Filderman AE Bruckner A Kacinski BM Deng N Remold HG Macrophage colony-stimulating factor (CSF-1) enhances invasiveness in CSF-1 receptor-positive carcinoma cell lines Cancer Res 1992 52 3661 6 1535551

[B2] Ide H Seligson DB Memarzadeh S Xin L Horvath S Dubey P Flick MB Kacinski BM Palotie A Witte ON Expression of colony-stimulating factor 1 receptor during prostate development and prostate cancer progression PNAS 2002 99 14404 9 10.1073/pnas.222537099 12381783 PMC137896

[B3] Kacinski BM Carter D Mittal K Yee LD Scata KA Donofrio L Chambers SK Wang KI Yang-Feng T Rohrschneider LR Ovarian adenocarcinomas express *fms*-complementary transcripts and *fms* antigen, often with coexpression of CSF-1 Am J Pathol 1990 137 135 47 1695482 PMC1877699

[B4] Kacinski BM Scata KA Carter D Yee LD Sapi E King BL Chambers SK Jones MA Pirro MH Stanley ER *FMS* (CSF-1 receptor) and CSF-1 transcripts and protein are expressed by human breast carcinomas *in vivo* and *in vitro* Oncogene 1991 6 941 52 1829808

[B5] Stanley ER Berg KL Einstein DB Lee PS Pixley FJ Wang Y Yeung YG Biology and action of colony–stimulating factor-1 Mol Reprod Dev 1997 46 4 10 10.1002/(SICI)1098-2795(199701)46:1<4::AID-MRD2>3.0.CO;2-V 8981357

[B6] Teitelbaum SL Ross FP Genetic regulation of osteoclast development and function Nat Rev Genet 2003 4 638 49 10.1038/nrg1122 12897775

[B7] Dai XM Ryan GR Hapel AJ Dominguez MG Russell RG Kapp S Sylvestre V Stanley ER Targeted disruption of the mouse colony-stimulating factor 1 receptor gene results in osteopetrosis, mononuclear phagocyte deficiency, increased primitive progenitor cell frequencies, and reproductive defects Blood 2002 99 111 20 10.1182/blood.V99.1.111 11756160

[B8] Chambers SK Role of CSF-1 in progression of epithelial ovarian cancer Future Oncol 2009 5 1429 1440 10.2217/fon.09.103 19903070 PMC2830097

[B9] Chambers SK Kacinski BM Ivins CM Carcanqiu ML Overexpression of epithelial macrophage colony-stimulating factor (CSF-1) and CSF-1 receptor: a poor prognostic factor in epithelial ovarian cancer, contrasted with a protective effect of stromal CSF-1 Clin Cancer Res 1997 3 999 1007 9815777

[B10] Chambers SK Wang Y Gertz RE Kacinski BM Macrophage colony-stimulating factor mediates invasion of ovarian cancer cells through urokinase Cancer Res 1995 55 1578 85 7882368

[B11] Toy EP Azodi M Folk NL Zito CM Zeiss CJ Chambers SK Enhanced ovarian cancer tumorigenesis and metastasis by the macrophage colony-stimulating factor Neoplasia 2009 11 136 44 19177198 10.1593/neo.81150 PMC2631138

[B12] Koths K Structure-function studies on human macrophage colony-stimulating factor (M-CSF) Mol Reprod Dev 1997 46 31 37 10.1002/(SICI)1098-2795(199701)46:1<31::AID-MRD6>3.0.CO;2-S 8981361

[B13] Ralph P Warren MK Lee MT Csejtey J Weaver JF Broxmeyer HE Williams DE Stanley ER Kawasaki ES Inducible production of human macrophage growth factor, CSF-1 Blood 1986 68 633 9 3488774

[B14] Price LK Choi HU Rosenberg L Stanley ER The predominant form of secreted colony stimulating factor-1 is a proteoglycan J Biol Chem 1992 267 2190 2199 1733926

[B15] Suzu S Ohtsuki T Makishima M Yanai N Kawashima T Nagata N Motoyoshi K Biological activity of a proteoglycan form of macrophage colony-stimulating factor and its binding to type V collagen J Biol Chem 1992 267 16812 16815 1512223

[B16] Zhou Y Yi X Stoffer JNB Bonafe N Gilmore-Hebert M McAlpine J Chambers SK The multifunctional protein glyceraldehydes-3-phosphate dehydrogenase is both regulated and controls colony-stimulating factor-1 messenger RNA stability in ovarian cancer Mol Cancer Res 2008 6 1375 1384 10.1158/1541-7786.MCR-07-2170 18708368 PMC2587019

[B17] Kim VN MicroRNA biogenesis: coordinated cropping and dicing Nat Rev Mol Cell Biol 2005 6 376 85 15852042 10.1038/nrm1644

[B18] Friedman RC Farh KK Burge CB Bartel DP Most mammalian mRNAs are conserved targets of microRNAs Genome Res 2009 19 92 105 18955434 10.1101/gr.082701.108 PMC2612969

[B19] Stark A Brennecke J Bushati N Russell RB Cohen SM Animal MicroRNAs confer robustness to gene expression and have a significant impact on 3'UTR evolution Cell 2005 123 1133 46 10.1016/j.cell.2005.11.023 16337999

[B20] Sugatani T Hruska KA Impaired micro-RNA pathways diminish osteoclast differentiation and function J Biol Chem 2009 284 4667 78 19059913 10.1074/jbc.M805777200 PMC2640963

[B21] Sorrentino A Liu CG Addario A Peschle C Scambia G Ferlini C Role of microRNAs in drug-resistant ovarian cancer cells Gynecol Oncol 2008 111 478 86 10.1016/j.ygyno.2008.08.017 18823650

[B22] Yang C Cai J Wang Q Tang H Cao J Wu L Wang Z Epigenetic silencing of miR-130b in ovarian cancer promotes the development of multidrug resistance by targeting colony-stimulating factor 1 Gynecol Oncol 2012 124 325 34 10.1016/j.ygyno.2011.10.013 22005523

[B23] Dweep H Sticht C Pandey P Gretz N miRWalk - Database: Prediction of possible miRNA binding sites by "walking" the genes of three genomes J Biomed Inform 2011 44 839 47 10.1016/j.jbi.2011.05.002 21605702

[B24] Griffiths-Jones S Saini HK van Dongen S Enright AJ miRBase: tools for microRNA genomics Nucleic Acids Res 2008 36 D154 8 10.1093/nar/gkn221 17991681 PMC2238936

[B25] Wang X miRDB: a microRNA target prediction and functional annotation database with a wiki interface RNA 2008 14 1012 7 10.1261/rna.965408 18426918 PMC2390791

[B26] Wang X El Naqa IM: Prediction of both conserved and nonconserved microRNA targets in animals Bioinformatics 2008 24 325 32 10.1093/bioinformatics/btm595 18048393

[B27] Krek A Grun D Poy MN Wolf R Rosenberg L Epstein EJ MacMenamin P da Piedade I Gunsalus KC Stoffel M Rajewsky N Combinatorial microRNA target predictions Nat Genet 2005 37 495 500 10.1038/ng1536 15806104

[B28] Chen K Rajewsky N Natural selection on human microRNA binding sites inferred from SNP data Nat Genet 2006 38 1452 6 10.1038/ng1910 17072316

[B29] Kertesz M Iovino N Unnerstall U Gaul U Segal E The role of site accessibility in microRNA target recognition Nat Genet 2007 39 1278 1284 10.1038/ng2135 17893677

[B30] Miranda KC Huynh T Tay Y Ang YS Tam WL Thomson AM Lim B Rigoutsos I A pattern-based method for the identification of MicroRNA binding sites and their corresponding heteroduplexes Cell 2006 126 1203 1217 10.1016/j.cell.2006.07.031 16990141

[B31] Lewis BP Burge CB Bartel DP Conserved seed pairing, often flanked by adenosines, indicates that thousands of human genes are microRNA targets Cell 2005 120 15 20 10.1016/j.cell.2004.12.035 15652477

[B32] Bonafe N Gilmore-Hebert M Folk N Azodi M Zhou Y Chambers SK Glyceraldehyde-3-phosphate dehydrogenase binds to the AU-rich 3’ untranslated region of colony-stimulating factor-1 (CSF-1) messenger RNA in human ovarian cancer cells: possible role in CSF-1 posttranscriptional regulation and tumor phenotype Cancer Res 2005 65 3762 3771 10.1158/0008-5472.CAN-04-3954 15867372

[B33] de Souza Rocha Simonini P Breiling A Gupta N Malekpour M Youns M Omranipour R Malekpour F Volinia S Croce CM Najmabadi H Diederichs S Sahin O Mayer D Lyko F Hoheisel JD Riazalhosseini Y Epigenetically deregulated microRNA-375 is involved in a positive feedback loop with estrogen receptor alpha in breast cancer cells Cancer Res 2010 70 9175 84 10.1158/0008-5472.CAN-10-1318 20978187

[B34] Maroney PA Chamnongpol S Souret F Nilsen TW Direct detection of small RNAs using splinted ligation Nat Protoc 2008 3 279 87 10.1038/nprot.2007.530 18274530

[B35] Smirnova L Grafe A Seiler A Schumacher S Nitsch R Wulczyn FG Regulation of miRNA expression during neural cell specification Eur J Neurosci 2005 21 1469 77 10.1111/j.1460-9568.2005.03978.x 15845075

[B36] Godlewski J Nowicki MO Bronisz A Williams S Otsuki A Nuovo G Raychaudhury A Newton HB Chiocca EA Lawler S Targeting of the Bmi-1 oncogene/stem cell renewal factor by microRNA-128 inhibits glioma proliferation and self-renewal Cancer Res 2008 68 9125 30 10.1158/0008-5472.CAN-08-2629 19010882

[B37] Huang J Wang Y Guo Y Sun S Down-regulated microRNA-152 induces aberrant DNA methylation in hepatitis B virus-related hepatocellular carcinoma by targeting DNA methyltransferase 1 Hepatology 2001 52 60 70 20578129 10.1002/hep.23660

[B38] Lui WO Pourmand N Patterson BK Fire A Patterns of known and novel small RNAs in human cervical cancer Cancer Res 2007 67 6031 43 10.1158/0008-5472.CAN-06-0561 17616659

[B39] Das S Foley N Bryan K Watters KM Bray I Murphy DM Buckley PG Stallings RL MicroRNA mediates DNA demethylation events triggered by retinoic acid during neuroblastoma cell differentiation Cancer Res 2010 70 7874 81 10.1158/0008-5472.CAN-10-1534 20841484 PMC2955783

[B40] Shtutman M Baig M Levina E Hurteau G Lim CU Broude E Nikiforov M Harkins TT Carmack CS Ding Y Wieland F Buttyan R Roninson IB Tumor-specific silencing of COPZ2 gene encoding coatomer protein complex subunit ς2 renders tumor cells dependent on its paralogous gene COPZ1 PNAS 2011 108 12449 54 10.1073/pnas.1103842108 21746916 PMC3145676

[B41] Leung AK Young AG Bhutkar A Zheng GX Bosson AD Nielsen CB Sharp PA Genome-wide identification of Ago2 binding sites from mouse embryonic stem cells with and without mature microRNAs Nat Struct Mol Biol 2011 8 237 44 21258322 10.1038/nsmb.1991 PMC3078052

[B42] Zhou Y Ferguson J Chang JT Kluger Y Inter- and intra-combinatorial regulation by transcription factors and microRNAs BMC Genomics 2007 8 396 10.1186/1471-2164-8-396 17971223 PMC2206040

[B43] Aravin AA Lagos-Quintana M Yalcin A Zavolan M Marks D Snyder B Gaasterland T Meyer J Tuschl T The small RNA profile during Drosophila melanogaster development Dev Cell 2003 5 337 50 10.1016/S1534-5807(03)00228-4 12919683

[B44] Guo H Ingolia NT Weissman JS Bartel D.P: Mammalian microRNAs predominantly act to decrease target mRNA levels Nature 2010 466 835 840 10.1038/nature09267 20703300 PMC2990499

[B45] Bazzini A Lee MT Giraldez AJ Ribosome profiling shows that miR-430 reduces translation before causing mRNA decay in Zebrafish Science 2012 336 233 237 10.1126/science.1215704 22422859 PMC3547538

[B46] Djuranovic S Nahvi A Green R miRNA-mediated gene silencing by translational repression followed by mRNA deadenylation and decay Science 2012 336 237 240 10.1126/science.1215691 22499947 PMC3971879

[B47] Huntzinger E Izaurralde E Gene silencing by microRNAs: contributions of translational repression and mRNA decay Nature Reviews: Genetics 2011 12 99 110 10.1038/nrg2936 21245828

[B48] Chen C Ridzon DA Broomer AJ Zhou Z Lee DH Nguyen JT Barbisin M Xu NL Mahuvakar VR Andersen MR Lao KQ Livak KJ Guegler KJ Real-time quantification of microRNAs by stem-loop RT-PCR Nucleic Acids Res 2005 33 e179 10.1093/nar/gni178 16314309 PMC1292995

[B49] Schmittgen TD Livak KJ Analyzing real-time PCR data by the comparative CT method Nature Protoc 2008 3 1101 1108 10.1038/nprot.2008.73 18546601

